# Validation of in-house liquid direct agglutination test antigen: the potential diagnostic test in visceral Leishimaniasis endemic areas of Northwest Ethiopia

**DOI:** 10.1186/s12866-020-01780-0

**Published:** 2020-04-15

**Authors:** Birhanu Ayelign, Mohammedamin Jemal, Markos Negash, Meaza Genetu, Tadelo Wondmagegn, Ayalew Jejaw Zeleke, Ligabaw Worku, Abebe Genetu Bayih, Girma Shumie, Sinknesh Wolde Behaksra, Tiruwork Fenta, Demekech Damte, Arega Yeshanew, Endalamaw Gadisa

**Affiliations:** 1 Department of Immunology and Molecular Biology, School of Biomedical and Laboratory Sciences, College of Medicine and Health Sciences, University of Gondar, Gondar, Ethiopia; 2 Department of Medical Parasitology, School of Biomedical and Laboratory Sciences, College of Medicine and Health Sciences, University of Gondar, Gondar, Ethiopia; 3 Armauer Hansen Research Institute, Addis Ababa, Ethiopia; 4 Leishmania Research and Treatment Center, University of Gondar Hospital, Gondar, Ethiopia

**Keywords:** Liquid direct agglutination test, Visceral leishmaniasis, North West Ethiopia

## Abstract

**Background:**

Visceral leishmaniasis in Ethiopia is a re-emerging threat to public health, with increased geographical distribution and number of cases. It is a fatal disease without early diagnosis and treatment; thus, the availability of affordable diagnostic tools is crucial. However, due to delays caused by import regulations, procurement and late delivery of imported test kits, accessibility remains a problem in the control program. Therefore, we aimed to produce and evaluate the performance of an in-house liquid (AQ) direct agglutination test (DAT) antigen.

**Result:**

The AQ-DAT was produced at the Armauer Hansen Research Institute, using *Leishmania donovani* strain (MHOM/ET/67/L82*)*. Sera from 272 participants; 110 microscopically confirmed cases of VL, 76 apparently healthy and 86 patients who had infectious disease other than VL were tested with AQ-DAT, and standard kits: Freeze-dried DAT (FD-DAT) and rK39. Taking microscopy as a gold standard; the sensitivity and specificity of the AQ-DAT were 97.3 and 98.8%, respectively. It had high degrees of agreement (k > 0.8), with a significant (*P* < 0.05) correlation compared to microscopy, FD-DAT, and rK39.

**Conclusion:**

Although further standardization is required, the in-house AQ-DAT could improve diagnostic accessibility, minimize intermittent stock outs and strengthen the national VL control program.

## Background

Visceral leishmaniasis, also known as kala-azar, is a neglected tropical disease. East Africa carries the second-highest burden of VL in the world. This region witnessed a re-emergence of VL, which makes it an increasing public health threat [1–3]. In the last few decades, VL outbreaks claimed hundreds of lives in the region both in the previously endemic and nonendemic areas [4–6]. In Ethiopia, over 33% of the total landmass are known to have the disease. Amhara, Tigray, Southern nation nationalities and peoples, Oromia and Somali region are at high risk of VL transmission in the country [7]. Visceral leishmaniasis in Ethiopia is caused by *Leishmania donovani (L. donovani),* and over 3.2 million people are estimated to be at risk with up to 5000 new cases a year [7, 8].

Visceral leishmaniasis is fatal without proper treatment in over 95% of the cases. The main drugs available for the treatment of VL are antimony compounds, sodium stibogluconate (SSG) and meglumine antimoniate (glucatim), Liposomal Amphotericin B (AmBisome), paromomycin and now the oral drug miltefosine. The current first line treatment for VL in Ethiopia is a combination of antimonial with aminoglycosides (SSG and Paromomycin), SSG or glucatim (Monotherapy) and paromomycin and Liposomal Amphotericin B (AmBisome) in special situations like pregnant women. Liposomal Amphotericin B (AmBisome), Miltefosine and Paromomycin (Aminosidine) are second-line treatment for primary VL. The drugs available are not only prohibitively expensive for the most affected, but also are associated with severe side effects. Thus, early and accurate diagnosis is crucial for VL treatment and control. The diagnostic approaches include the direct methods; microscopy, culture and polymerase chain reaction (PCR), and the indirect ones, the patient produce an immune response to the *L. donovani*, the agent causing of VL in East Africa, are direct agglutination test (DAT), rK39 immuno-chromatographic test (ICT), and indirect fluorescence antibody test (IFAT) [9]. Most of the direct methods involve invasive and technically demanding sampling procedures and/or processing steps that need specialized training and expensive set-ups. Thus, their use in most endemic settings are hardly possible. Of the indirect method, DAT and rK39-ICT are less technically demanding and involve minimally invasive sampling procedures. They are the most widely validated tools in the Ethiopian endemic area [10–13]. Among them, DAT was shown to be more sensitive, specific and reproducible [12, 13]. The production technique of the DAT antigen is not patented, and elsewhere the use of local strains of VL causing strain makes it even more sensitive and specific [14, 15]. Yet, the national program is highly dependent on foreign aid to purchase DAT and this leads for unnecessary delays due to import regulations and processes. As a result, improving accessibility remains a challenge. Thus, local production of aqueous DAT antigen could increase user-friendliness and avoids/minimize stock-outs.

## Result

### Sociodemographic characteristics of study participants

We used a serum sample for a total of 272 study subjects (110 VL patients and 162 controls) to evaluate the reliability and validity of the DAT antigen developed in-house for the diagnosis of VL. Among these 185 (68.0%) were males, 170 (62.5%) were within the age group 15–30 years, and 51 (18.75%) were agricultural laborers (Table 1).

**Table 1:** Sociodemographic characteristics of study participants (*N* = 272)

Variables	Frequency, N (%)		
	Endemic healthy control (*N* = 76)	Other than VL disease (*N* = 86)	VL (*N* = 110)
Sex			
Male	45 (59)	59 (69)	81 (74)
Female	31 (41)	27 (31)	29 (26)
Age			
< 15	0 (0)	7 (8.1)	0 (0)
15–30	58 (76.3)	46 (53.5)	66 (60)
30–45	16 (21.1)	23 (26.7)	43 (39.1)
45–55	4.2 (2.6)	9 (10.5)	1 (0.9)
> 55	0 (0)	1(1.2	0 (0)
Occupation			
Own agriculture	20 (26)	17 (20)	30 (27.2)
Agricultural laborer	11 (15)	2 (2)	38 (34.5)
Civil servants	23 (30)	19 (22)	13 (11.8)
Other	22 (29)	48 (56)	29 (26.4)
Educational status			
Illiterate	23 (30.3)	29 (33.7)	49 (44.5)
Primary school	21 (27.6)	17 (19.8)	39 (35.5)
Above Secondary	16 (21)	31 (36)	13 (11.8)
Degree and above	16 (21)	9 (10.5)	09 (8.2)
Clinical presentation			
Fever > 2 week	0 (0)	43 (26.5)	87 (79)
Splenomegaly	0 (0)	04 (4.7)	97 (88.5)
Weight loss	0 (0)	11 (13)	76 (69)
Hepatosplenomegaly	0 (0)	09 (10.)	54 (49)

### Performance of AQ-DAT, FD-DAT antigen and rK39-ICT

Out of the 110 microscopically confirmed cases of VL, 107 (97.1%), 109 (99.1%), and 106 (96.4%) were tested positive with AQ-DAT, FD-DAT, and rK39-ICT tests, respectively. The cross reactivity of sera from CL (*N* = 15) cases were the highest for rK39-ICT, 7(46.7%), followed by FD-DAT, 4(26.7%). AQ-DAT showed the least cross reactivity, 2 (13.3%). In addition, 13.3% (2/15) 2.44% (1/41) and 6.7% (1/15) of serum sample from schistosomiasis, malaria and tuberculosis patients were tested positive for only rK39-ICT, respectively (Table 2).

**Table 2:** Comparison of the performance of AQ-DAT, FD-DAT and rK39-ICT for the diagnosis of visceral leishmaniasis

Source of Serum	AQ- DAT		FD-DAT		rK39-ICT	
	Pos N (%)	Neg N (%)	Pos N (%)	Neg N (%)	Pos N (%)	Neg N (%)
VL cases	107 (97.1)	3 (2.7)	109 (99.1)	1 (0.9)	106 (96.4)	4 (3.6)
CL Cases	2 (13.3)	13 (86.7)	4 (26.7)	11 (73.3)	7 (46.7)	8 (53.3)
Schistosomiasis	0 (0)	15 (100)	0 (100)	15 (100)	2 (13.3)	13 (86.7)
Malaria	0 (0)	41 (100)	0 (0)	41 (100)	1 (2.44)	40 (100)
Tuberculosis	0 (0)	15 (100)	0 (100)	15 (100)	1 (6.7)	14 (93.3)
Apparently Healthy	0 (0)	76 (100)	0 (0)	76 (100)	0 (0)	76 (100)

N.B Pos- positive, Neg- negative.

**Fig. 1:**
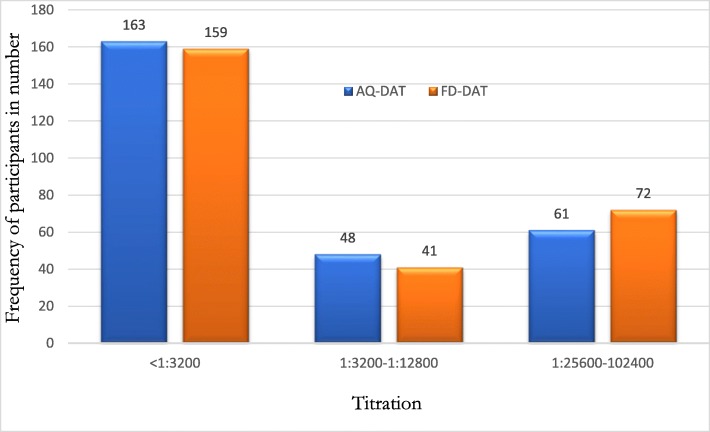
Measure of agreement between the in-house AQ-DAT with FD-DAT considers the cutoff titer category value as per the ITMA-DAT recommendation

Taking microscopy as the Gold standard, the sensitivity of the AQ-DAT, FD-DAT and rK39-ICT with 95% confidence interval were 97.3% (93.7–98.7), 99.1% (95.6–100) and 96.4% (91.8–98.7), respectively; while the specificity of the AQ-DAT, FD-DAT and rK39-ICT were 98.8% (96.4–99.8), 97.5% (95.2–98.1) and 93.2% (90.1–94.8), respectively. Both AQ-DAT and FD-DAT showed comparable sensitivity and specificity, while rK39-ICT rapid tests showed slightly low validity of the test (Table 2). The in-house AQ-DAT showed a high level of agreement with both FD-DAT (*k = 0.962, P < 0.00)* and rK39-ICT (k = 0.895, P < 0.00). While 72/113 (64%) and 61/109 (56%) were positive at high titer (> 1:25600) with FD-DAT and AQ-DAT, respectively (Fig. 1).

## Discussion

The definitive diagnosis of VL has crucial importance not only because it is almost always fatal if left untreated, but also the delay in diagnosis has implications for the transmission and reduces cure rates [8, 16]. Moreover, the high cost and severe side effects associated with the available chemotherapeutic options made the value for prompt and accurate diagnosis unquestionable [3, 17, 18]. However, the VL endemic East African countries, including Ethiopia lack sufficient capacity and resource for the purchase of diagnostic supplies, thus their control programs are donor dependent. The national neglected tropical disease programs, Ethiopian federal ministry of health recommended rK39-ICT at the primary health care center and DAT, and Microscopy at district and tertiary hospitals as a diagnostic tool for VL [19]. Yet accessibility is limited due to delays related to import regulations and processes, late ordering, intermittent stock outs even in the referral setups. Thus, in this study, we produced whole cell DAT antigen in liquid using MHOM/ET/67/L82 *L. donovani* strain and assessed performance comparing it with validated commercial kits; FD-DAT (ITMA- DAT/VL, Belgium) and rK39-ICT (InBios International Kalazar DetectTM Rapid test kit, The Netherlands).

Our in-house AQ-DAT had a sensitivity (97.3%) comparable to FD-DAT (99.1%) and rK39-ICT (96.5%), taking microscopy as gold standard. This is similar to a study done in Sudan in which AQ-DAT, FD-DAT, and rK39 showed a sensitivity of 99, 95.8, and 79.2%, respectively [14]. Similarly, studies conducted in Brazil and Sudan documented better sensitivity of FD-DAT (98–100%) compared to rK39-ICT (85.7–90%) [20, 21]. In contrast, another study from the Northeast of Sudan showed, lower sensitivity for FD-DAT (84%) compared with rK39-ICT (93%) [22]. Overall, the observed differences in sensitivity among studies could be due to the strain variation that affects the gene expression level of rK39 protein. Moreover, it might be related with the difference in manufacturer of the rK39-ICT test strip and the diversity in host immune response.

The specificity of the in-house AQ-DAT, FD-DAT and rK39-ICT were 98.8, 97.5 and 93.2%, respectively, which is in line with findings of the studies done in Sudan which revealed 100, 100 and 97.6%, respectively [14, 21]. The AQ and FD-DAT, also showed similar specificity with the findings reported from the United Kingdom, Brazil and Sudan [20, 21, 23]. However, rK39-ICT in our study was higher than finding from Brazil (82%), and lower than finding from Ethiopia, Brazil, Sudan and United Kingdom (99, 100, 100%, respectively) [10, 20, 23].

In the present study, the comparison among AQ-DAT, FD-DAT and rK39-ICT found two to 10 controls as positive. The AQ-DAT, FD-DAT and rK39-ICT resulted in cross-reactivity with serum samples of parasitologically confirmed cases of CL 2/15 (1.2%), 4/15 (2.5%) and 7/15 (6.8%), respectively; rK39-ICT also reacted with 4 out of 15 schistosomiasis positive serum samples. It is plausible to attribute this to the genetic similarity of CL and VL causative agents of the same genus [24–26]. The rK39-ICT reaction with sera from schistosomiasis patients. It is worth notice in diagnosing migrant laborers from endemic areas, to prevent unwanted complications with anti-leishmanial treatment through better diagnostic tool.

All apparently healthy control groups were negative with AQ-DAT, FD-DAT and rK39-ICT, and it is in line with a study in Sudan [27]. In contrast, a study conducted in Brazil and Sudan; have a cross-reaction with healthy control [21, 28]. Herein, we observed that AQ-DAT and FD-DAT are more specific and able to correctly identify the study subjects than rK39-ICT. Although microscopy is taken as a gold standard [29, 30] in this study, positive response to specific anti-leishmanial treatment has also been reported in VL suspects (with un-confirmed infection) that tested positive in the DAT. Moreover, in the present study, high specificity could be due to the enrollment of control groups from VL non-endemic area.

The reproducibility of the in-house AQ antigen compared to FD-DAT and rK39-ICT (k = *0.962, P* < 0.00) and K = *0.895,* P < 0.00, respectively). The accuracy of the rK39-ICT was comparable to a study conducted other part of in Ethiopia (90.6%, K = 0.81; *P* < 0.05). the current findings demonstrates a substantial level of agreement between FD-DAT in this study (k = 0.962, *P* < 0.00) and study conducted in Ethiopia (87.7% with k = 0.75, P < 0.05) [12]. Hence, its reliability and the sustainable access of AQ-DAT might have advantages for use in peripheral health services compared with the current rK39-ICT and FD-DAT. This study did not assess the variations in the performance of the in-house liquid DAT antigen based on its stability by storing it after various period of time at different temperature. In this study, we assessed the diagnostic performance of AQ-DAT antigen with respect to FD-DAT and rK39, and found an encouraging outcome. The production and distribution of in-house DAT antigen to the end-users in different parts of Ethiopian Health Services requires involvement of commercial companies and/or research institutes. Currently, we plan to secure the implementation of AQ-DAT antigen production at larger scale as well as testing batch-to-batch variability and stability with the support and collaboration of a research institute (AHRI) and academic-research institute (University of Gondar).

## Conclusion

Overall, AQ-DAT demonstrated comparable performance compared to FD-DAT and rK39-ICT in diagnosing VL patients. Thus, the AQ-DAT merits GCLP standard production with wider evaluation. Doing so, we believe this would make access to VL diagnosis more equitable and enable to build self-sustained programs.

## Methods

### Study population, design and period

Volunteered and consecutive microscopically confirmed: VL (*N* = 110), pulmonary tuberculosis (*N* = 15), malaria (*N* = 41), schistosomiasis (N = 15) and cutaneous leishmaniasis (CL, N = 15) cases were recruited at the University of Gondar Hospital (UoGH) Leishmaniasis Research and Treatment center (LRTC) and the outpatient ward from June 2016 up to August 2018. Also, 76 apparently healthy individuals from areas not known to be VL endemic blood donors were enrolled.

A questionnaire was used to capture sociodemographic, previous medical and travel history related to VL. Apparently healthy and non-VL patients with a reported history of VL and/or travel history to VL endemic area(s) were excluded from the study. Serum was separated from ~ 8 mL of venous blood and stored at − 20 °C until transported to AHRI.

### Production of liquid (AQ) direct agglutination test (DAT) antigen

The AQ-DAT was produced using the MHOM/ET/67/L82 *L. donovani* strain at AHRI. Antigen preparation was done according to the improved protocol described by el Harith (personal communication, Ahfad University for Women, Khartoum, Sudan). The strain was mass cultured in liver infusion tryptose (LIT) media supplemented with 0.5% hemin (Oxoid Ltd., London, UK), 10% Fetal bovine serum (FBS, Thermo Fisher Scientific, Gibco, Carlsbad, California), 1% Penicillin-Streptomycin (Thermo Fisher Scientific Inc., USA) for 1 week. Logarithmic stage promastigotes were harvested by centrifugation at 3000 RPM (Megafuge, Thermo Fisher Scientific Inc., USA) at 4 °C for 10 min. The harvested promastigotes were washed three times with Lock’s solution at the same condition above and then treated twice with 0.6% 2-Mercaptoethanol in Lock’s solution and incubate at 37 °C for 1 h. Then, promastigotes were washed three times with Lock’s solution at the same centrifugation speed at 4 °C. Followed by fixation with 3% (V/V) Formaldehyde in Lock’s solution kept overnight at 4 °C.

After overnight incubation, the sediment washed three times by sodium-citrate-saline (0.15 M NaCl and 0.05 M sodium citrate) at 3000 RPM at 4 °C for 10 min. Subsequently re-suspended in 100 mL of sodium-citrate-saline solutions (0.15 M NaCl and 0.05 M sodium citrate) and stained in 0.2% Coomassie-brilliant blue (Fisher Scientific: Janssen Pharmaceutical, Geel, Belgium). After 3 h staining and checking microscopically for the absence of any aggregation and consistency of staining, the excess stain was washed, 2–3 times, in sodium-citrate-saline solutions (0.15 M NaCl and 0.05 M sodium citrate) until the supernatant became clear. According to the final Parasite Cell pellet Volume (PCV), the sediment was re-suspended in 1.2% (V/V) formal-citrate solution (3% formaldehyde in sodium-citrate-saline) and kept in a magnetic stir for 1 h. Optimization of the concentration of antigen (stained promastigotes) was done by counting cells using Neubauer improved hemocytometer-having a depth of 0.02 mm and 0.0025mm^2^. Usually, 1 mL PCV is expected from one litter culture, which will be resuspended in 100 mL 1.2% (V/V) formal-citrate solution and the concentration was adjusted to 5 × 10^7^ parasites/mL. The prepared antigen was kept at 2-8 °C until used. Positive and negative sera references have been including the maximum number of days Sera have been measured in 2-fold serial dilutions between 1:100 and 1:102,400. Serial dilutions showing ≥ 1:3200 titrations were considered positive for VL.

### Direct agglutination and rK39 tests

Sera from participants were tested with the rK39-ICT kit (Lot number WM1264 with expired date 12/2019, INBios international Kalazar Detect™, The Netherlands), and FD-DAT (Lot number AHRI-2017-1B with expired date 01/11/2018, ITMA-DAT, Belgium) as per the suppliers’ recommendations. All samples were tested with the AQ-DAT, FD-DAT and rK39-ICT test and read by the same person. Moreover, all sera were tested at AHRI without prior knowledge of the sample results. The in-house AQ-DAT was used following the same procedure as for the commercial FD-DAT. In brief:

*DAT:* the *FD-*DAT antigen was reconstituted according to the manufacturer’s instructions. T*wo*fold dilution series of the sera were made, starting at a dilution of 1:100 and going up to a maximum serum dilution of 1:102.400. Fifty μL DAT antigens were added to each well containing 50 μl diluted serum (1 μL patient serum in 49 μL gelatin-saline diluents containing 0.6% 2-Mercaptoethanol). After 18 h of incubation at room temperature samples with titer > = 1:3200 were read positive. Positive and negative controls (ITMA-DAT) were included in every 5th plat. For AQ-DAT, all procedures were the same; the effort was made to adjust the antigen concentration; roughly around 5 × 10^7^ parasites per mL, after counting the promastigotes in the FD-DAT. We adjusted it by counting Cell to optimize the concentration of the antigen using Neubauer improved hemocytometry having depth 0.02 mm and 0.0025mm^2^.

rK39-ICT: A 20 μL serum sample was added, followed by 2–3 drops of chase buffer solution to the absorbent pad and results were read after 10–20 min. Results were interpreted as positive when both control and test lines appear; negative when the only control line appears; invalid when no control line appears. Invalid results were repeated as per the manufacturer’s recommendation.

### Quality assurance

A blood specimen was collected using appropriate tube and transported to the testing lab according to standardized procedures. During the storage of sample temperature monitoring of all incubators, fridges, and freezers were assessed regularly by using calibrated thermometers. All assays were performed according to the manufacturer’s instructions and standard operation procedures (SOPs) were strictly followed. Culture media preparation and sterility testing were conducted according to the SOPs and performance of the media were tested with known reference *L. donovani* promastigote. The qualities of all commercial test kits were evaluated by using a known positive and negative serum before the actual test performed. Laboratory tests were performed and interpreted blindly without prior knowledge of the previous result.

### Statistical analysis

The data were double entered and cleaned using Epi Data 3.1 (Jens M. Lauritsen & Michael Bruus) and transferred to SPSS version 20 (IBM, New York, and U.S). Java Stat-two-way contingency table analysis software, (http://statpages.org/ctab2x2.html) was used to calculate sensitivity, specificity, and kappa values. Kappa agreement between AQ-DAT with FD-DAT and rK39-ICT with a 95% confidence interval were determined (0.81 to 1.0 = almost perfect agreement, 0.61–0.8 = substantial, 0.41–0.6 = moderate, 0.21–0.4 = fair, 0.2–0 = slight, < 0.0 = poor) [31].

## Data Availability

Data support these findings are contained within the manuscript and will share upon request to the corresponding author.

## Abbreviations

AHRI: Armauer Hansen Research Institute
DAT: Direct agglutination test
FD: Commercially Available Freeze-Dried
ITMA: Institute of Tropical Medicine in Antwerp
LIT: Liver infusion tryptose
rK39 ICT: Recombinant K protein immunochromatographic test
AQ: Aqueous antigen
UoGH: University of Gondar Hospital
VL: Visceral Leishmaniasis
